# Investigating causal associations among gut microbiota, gut microbiota-derived metabolites, and gestational diabetes mellitus: a bidirectional Mendelian randomization study

**DOI:** 10.18632/aging.204973

**Published:** 2023-08-23

**Authors:** Xinrui Wu, Dihui Lin, Qi Li, Jiawang Cai, Houxiang Huang, Tianyu Xiang, Hongzhuan Tan

**Affiliations:** 1 School of Medicine, Jishou University, Jishou, China; 2 Xiangya School of Public Health, Central South University, Changsha, China; 3 Xiangxi Center for Disease Control and Prevention, Jishou, China

**Keywords:** gestational diabetes mellitus, gut microbiota, gut microbiota-derived metabolites, Mendelian randomization, causal association

## Abstract

Background: Previous studies have shown that gut microbiota (GM) and gut microbiota-derived metabolites are associated with gestational diabetes mellitus (GDM). However, the causal associations need to be treated with caution due to confounding factors and reverse causation.

Methods: This study obtained genetic variants from genome-wide association study including GM (*N* = 18,340), GM-derived metabolites (*N* = 7,824), and GDM (5,687 cases and 117,89 controls). To examine the causal association, several methods were utilized, including inverse variance weighted, maximum likelihood, weighted median, MR-Egger, and MR.RAPS. Additionally, reverse Mendelian Randomization (MR) analysis and multivariable MR were conducted to confirm the causal direction and account for potential confounders, respectively. Furthermore, sensitivity analyses were performed to identify any potential heterogeneity and horizontal pleiotropy.

Results: Greater abundance of *Collinsella* was detected to increase the risk of GDM. Our study also found suggestive associations among *Coprobacter*, *Olsenella*, *Lachnoclostridium*, *Prevotella9*, *Ruminococcus2*, *Oscillibacte*, and *Methanobrevibacter* with GDM. Besides, eight GM-derived metabolites were found to be causally associated with GDM. For the phenylalanine metabolism pathway, phenylacetic acid was found to be related to the risk of GDM.

Conclusions: The study first used the MR approach to explore the causal associations among GM, GM-derived metabolites, and GDM. Our findings may contribute to the prevention and treatment strategies for GDM by targeting GM and metabolites, and offer novel insights into the underlying mechanism of the disease.

## INTRODUCTION

Gestational diabetes mellitus (GDM) is a dangerous gestational complication affecting 5-20% of pregnant women, and its prevalence is on the rise [1]. GDM increases the probability of adverse pregnancy outcomes, such as preterm birth, fetal malformation, and macrosomia [2–4]. It is also associated with maternal health problems, including gestational hypertension, postpartum hemorrhage, and dystocia in mothers [2, 5]. Additionally, offspring of mothers with GDM face an elevated risk of diabetes, hypertension, obesity, and coronary heart disease [3, 6]. Although various mechanisms including *β*-cell dysfunction, chronic insulin resistance, adipose tissue inflammation, and oxidative stress have been studied, the explicit pathogenesis of GDM remains unclear [7, 8].

Gut microbiota (GM) and microbial metabolites play important roles in maintaining host physiology and homeostasis and have been observed to change significantly during gestation. Mounting evidence demonstrated the gut microbiota dysbiosis in GDM patients [9], however, such findings differed across studies. Unlike other findings, Zhong et al. reported that *Coprococcus* decreases in GDM patients [10]. Karlsson et al. and Wu et al. found that *Clostridium* is a risk factor [11, 12], while Allin et al. reported the opposite result [13]. The interaction between the host and microbiota is primarily mediated by GM-derived metabolites. Numerous observational studies have indicated the association between GM-derived metabolites and GDM [14]. However, caution should be exercised in interpreting the association due to confounding factors and the complex environment of the human intestine, as well as the limitations of the observational study design.

Mendelian randomization (MR) is a useful approach to detect and quantify the causal effect of exposures on outcomes by using genetic variants as instrumental variables (IVs) [15]. Since alleles are randomly assigned from parents to offspring, freely combined, and genotypes remain stable after birth. MR, similar to a randomized controlled trial (RCT), can help minimize biases caused by traditional confounders (e.g., environmental exposures, demographic characteristics, and dietary habits) and reverse causation [16, 17]. Many studies have used MR analysis to explore the correlation among GM, GM-derived metabolites and complex human diseases such as metabolic diseases [18], neurodegenerative diseases [19], and adverse pregnancy outcomes [20]. Therefore, our study conducted bidirectional MR analyses using summary statistics from genome-wide association studies (GWAS) to investigate the causal relationship between GM, GM-derived metabolites, and GDM. This analysis may offer new insights into the underlying mechanism of GDM.

## RESULTS

A total of 7,121 SNPs associated with 119 bacterial genera were included for GM instruments, and 9,270 SNPs associated with 81 traits were identified for GM-derived metabolite instruments. Details of selected IVs in this study were shown in Supplementary Tables 1, 2.

### Associations between GM and GDM

**Figure 1:**
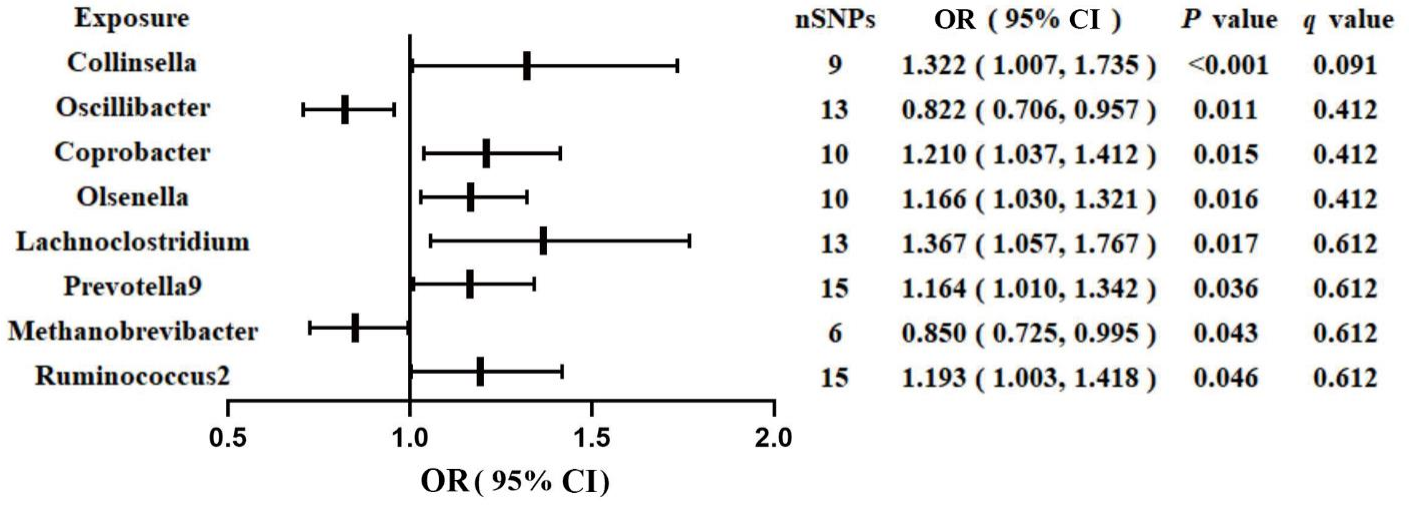
**Associations of genetically predicted gut microbiota with risk of GDM using IVW method.** OR, odds ratio; Cl, confidence interval.

**Figure 2:**
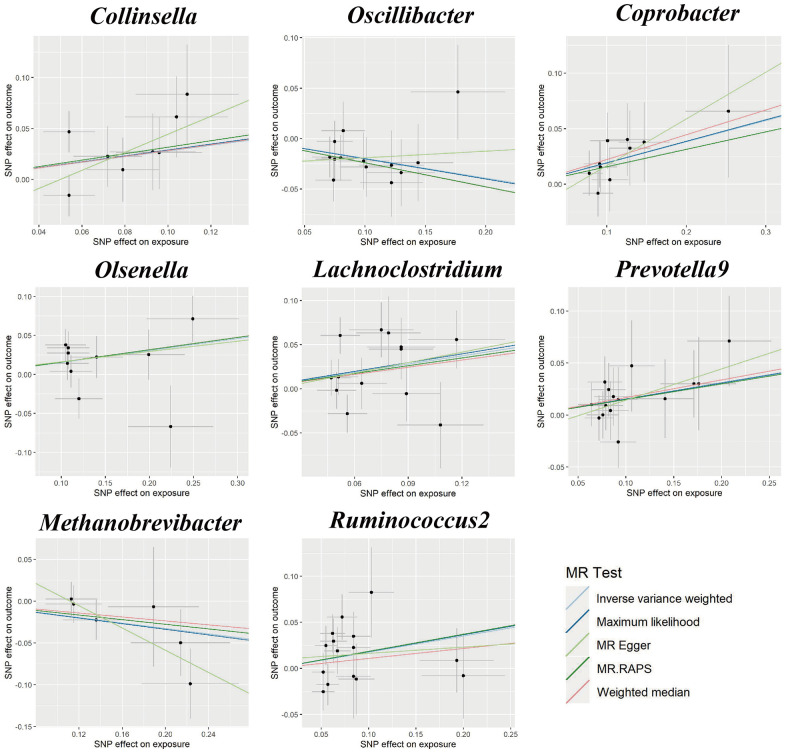
Scatter plots for the causal association between gut microbiota and GDM.

Figure 1 shows the results obtained using the IVW method at a significance threshold of *P* < 0.05. We identified significant positive associations between increases in *Collinsella* (OR, 1.322; 95%CI, 1.007-1.735; *P* < 0.001), *Coprobacter* (OR, 1.210; 95%CI, 1.037-1.412; *P* = 0.015), *Olsenella* (OR, 1.166; 95%CI, 1.030-1.321; *P* = 0.015), *Lachnoclostridium* (OR, 1.367; 95%CI, 1.057-1.767; *P* = 0.017), *Prevotella9* (OR, 1.164; 95%CI, 1.010-1.342; *P* = 0.036), *Ruminococcus2* (OR, 1.193; 95%CI, 1.003-1.418; *P* = 0.046) and a higher risk of GDM. Conversely, genetically increased levels of *Oscillibacter* (OR, 0.822; 95%CI, 0.706-0.957; *P* = 0.011) and *Methanobrevibacter* (OR, 0.850; 95%CI, 0.725-0.995; *P* = 0.043) were associated with a protective effect on GDM. Even after correcting for multiple comparisons, we observed a significant causal effect of increased *Collinsella* on the risk of GDM (*q* = 0.091). The *F*-statistics ranged from 20.39 to 336.56 in the aforementioned results, excluding the bias from weak instrumental variables. Additionally, we identified causal associations between GM and GDM risk in more than three MR methods, including IVW, MaxLik, WM, MR-Egger regression, and MR.RAPS (Table 1 and Figure 2).

**Table 1:** MR analyses of gut microbiota on GDM by different methods.

**Exposure**	***F* statistics**	**Inverse variance weighted**			**Maximum likelihood**			**Weighted median**			**MR.RAPS**			**MR Egger**	
		**OR (95%CI)**	***P***		**OR (95%CI)**	***P***		**OR (95%CI)**	***P***		**OR (95%CI)**	***P***		**OR (95%CI)**	***P***
*Collinsella*	28.40	1.322 (1.007,1.735)	<0.001		1.337 (1.023,1.746)	<0.001		1.329 (0.916,1.928)	0.010		1.373 (1.082,1.742)	0.012		2.423 (0.902,6.506)	0.123
*Oscillibacter*	27.30	0.822 (0.706,0.957)	0.011		0.819 (0.701,0.956)	0.014		0.788 (0.644,0.963)	0.022		0.847 (0.734,0.976)	0.022		1.068 (0.603,1.890)	0.830
*Coprobacter*	26.02	1.210 (1.037,1.412)	0.015		1.213 (1.036,1.421)	0.020		1.250 (1.023,1.527)	0.030		1.170 (1.014,1.350)	0.031		1.522 (0.852,2.717)	0.194
*Olsenella*	20.39	1.166 (1.029,1.321)	0.017		1.172 (1.046,1.314)	0.017		1.173 (1.006,1.367)	0.041		1.172 (1.042,1.319)	0.013		1.142 (0.747,1.745)	0.573
*Lachnoclostridium*	24.83	1.367 (1.057,1.767)	0.017		1.393 (1.114,1.741)	<0.001		1.313 (0.953,1.810)	0.100		1.340 (1.085,1.655)	0.010		1.485 (0.596,3.700)	0.412
*Prevotella9*	336.56	1.164 (1.010,1.342)	0.036		1.168 (1.011,1.349)	0.036		1.184 (0.981,1.430)	0.081		1.162 (1.013,1.334)	0.033		1.350 (0.893,2.043)	0.183
*Methanobrevibacter*	27.75	0.850 (0.725,0.995)	0.043		0.845 (0.717,0.995)	0.040		0.889 (0.718,1.101)	0.280		0.871 (0.753,1.009)	0.070		0.514 (0.283,0.933)	0.301
*Ruminococcus2*	21.13	1.193 (1.003,1.418)	0.046		1.201 (1.009,1.428)	0.045		1.114 (0.865,1.436)	0.410		1.204 (1.008,1.437)	0.043		1.071 (0.698,1.643)	0.760

OR, odds ratio; CI, confidence interval; P, P value; MR, mendelian randomization; MR.RAPS, mendelian randomization robust adjusted profile score; GDM, gestational diabetes mellitus.

Cochran’s Q statistics showed no significant heterogeneity in selected IVs (*P* > 0.05 in IVW and MR-Egger methods, Supplementary Table 3). Both the MR-Egger intercept and the MR-PRESSO global test confirmed there are no significant directional horizontal pleiotropy (*P* > 0.05, Supplementary Table 3). Additionally, the leave-one-out analysis revealed that there are no outlier IVs that would have a significant impact on the result (Supplementary Figure 1).

All methods in reverse MR analysis showed no causal relationship from GDM to GM (*P* > 0.05, Supplementary Table 4). The sensitivity analyses including Cochran’s Q statistics, MR-Egger intercept, MR-PRESSO global test, and the leave-one-out analysis demonstrated the robustness of the reverse MR results (Supplementary Table 5 and Supplementary Figure 2).

The MVMR results demonstrated that, even after adjusting for confounding factors, including BMI (OR, 1.470; 95%CI, 1.137-1.901; *P* = 0.003), alcohol drinking (OR, 1.486; 95%CI, 1.116-1.980; *P* = 0.006), smoking (OR, 1.589; 95%CI, 1.192-2.119; *P* = 0.001), and hypertension (OR, 1.286; 95%CI, 1.034-1.599; *P* = 0.023), the genus *Collinsella* maintained its causal association with the risk of GDM and exhibited a more significant effect compared to the univariable MR analysis. Detailed MVMR results for other GM on GDM were shown in Table 2.

**Table 2:** Multivariable MR analyses of gut microbiota on GDM after adjusting confounding factors.

**Exposure**	**BMI**			**Alcohol drinking**			**Smoking**			**Hypertension**	
	**OR (95%CI)**	***P***		**OR (95%CI)**	***P***		**OR (95%CI)**	***P***		**OR (95%CI)**	***P***
*Collinsella*	1.470(1.137,1.901)	0.003		1.486(1.116,1.980)	0.006		1.589(1.192,2.119)	0.001		1.286(1.034,1.599)	0.023
*Oscillibacter*	1.265(1.190,1.344)	<0.001		1.209(1.093,1.337)	<0.001		1.144(1.036,1.265)	0.008		1.217(1.120,1.323)	<0.001
*Coprobacter*	1.378(0.881,2.154)	0.160		1.330(0.996,1.777)	0.053		1.319(1.029,1.689)	0.029		1.119(0.723,1.732)	0.615
*Olsenella*	0.899(0.779,1.039)	0.150		0.838(0.722,0.972)	0.020		0.783(0.604,1.015)	0.065		0.817(0.666,1.002)	0.053
*Lachnoclostridium*	1.200(0.974,1.477)	0.086		1.140(0.990,1.313)	0.068		1.175(1.021,1.352)	0.025		1.148(0.978,1.349)	0.092
*Prevotella9*	0.798(0.735,0.867)	<0.001		0.849(0.756,0.954)	0.006		0.831(0.737,0.937)	0.003		0.814(0.716,0.926)	0.002
*Methanobrevibacter*	1.138(1.033,1.255)	0.009		1.167(1.040,1.309)	0.008		1.150(1.042,1.269)	0.006		1.157(1.043,1.283)	0.006
*Ruminococcus2*	1.197(0.990,1.447)	0.064		1.263(1.023,1.599)	0.030		1.207(1.022,1.426)	0.027		1.096(0.875,1.374)	0.424

OR, odds ratio; CI, confidence interval; P, P value; MR, mendelian randomization; GDM, gestational diabetes mellitus; BMI, body mass index.

### Associations between GM-derived metabolites and GDM

**Figure 3:**
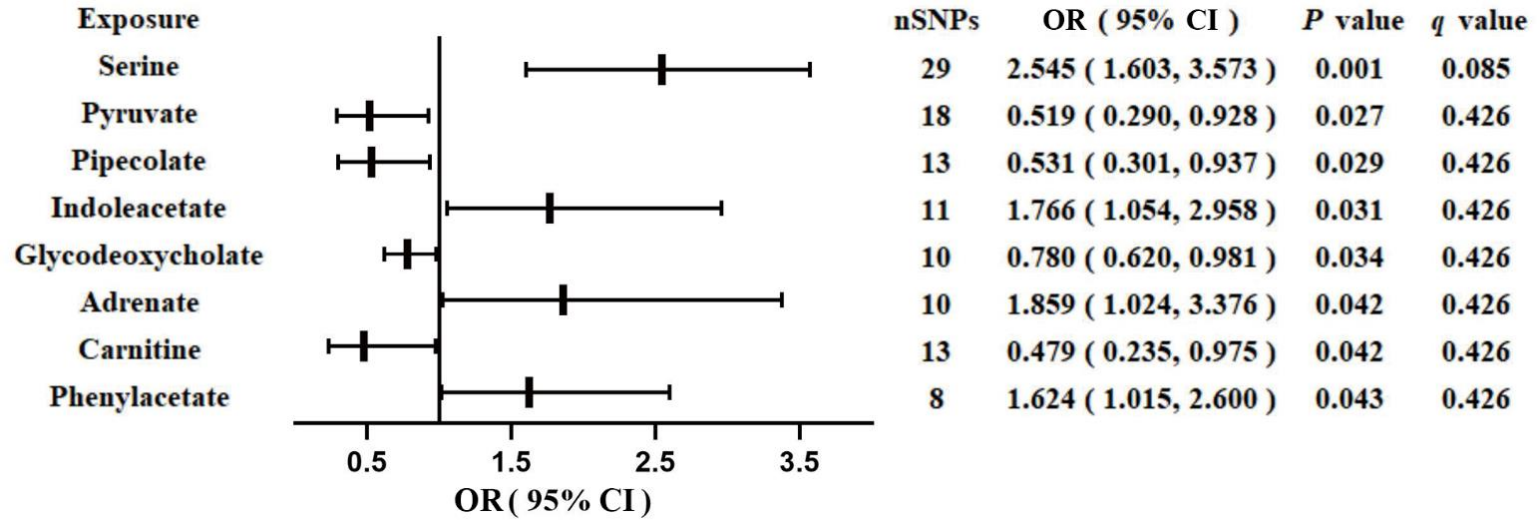
**Associations of genetically predicted gut microbiota-derived metabolites with risk of GDM using IVW method.** OR, odds ratio; CI, confidence interval.

We identified eight GM-derived metabolites that showed suggestive associations with GDM (*P* < 0.05, *q* > 0.1; Figure 3). Specifically, serine (OR, 2.545; 95%CI, 1.603-3.573; *P* = 0.001), indoleacetate (OR, 1.766; 95%CI, 1.054-2.958; *P* = 0.031), adrenate (OR, 1.859; 95%CI, 1.024-3.376; *P* = 0.042), and phenylacetate (OR, 1.624; 95%CI, 1.015-2.600; *P* = 0.043) were identified as risk factors for GDM, whereas pyruvate (OR, 0.519; 95%CI, 0.290-0.928; *P* = 0.027), pipecolate (OR, 0.531; 95%CI, 0.301-0.937; *P* = 0.029), glycodeoxycholate (OR, 0.780; 95%CI, 0.620-0.981; *P* = 0.034), and carnitine (OR, 0.479; 95%CI, 0.235-0.975; *P* = 0.042) were identified as protective factors for GDM. The *F*-statistics ranged from 15.70 to 65.99 in the aforementioned results, excluding the bias from weak instrumental variables. Furthermore, we explored causal associations between the GM-derived metabolites and GDM risk using more than three MR methods (Table 3 and Supplementary Figure 3). The sensitivity analyses demonstrated the robustness of the MR results (Supplementary Table 3 and Supplementary Figure 3, 4).

**Table 3:** MR analyses of gut microbiota-derived metabolites on GDM by different methods.

**Exposure**	***F* statistics**	**Inverse variance weighted**			**Maximum likelihood**			**Weighted median**			**MR.RAPS**			**MR Egger**	
		**OR (95%CI)**	***P***		**OR (95%CI)**	***P***		**OR (95%CI)**	***P***		**OR (95%CI)**	***P***		**OR (95%CI)**	***P***
Serine	34.57	2.545 (1.603,3.573)	0.001		2.403 (1.651,3.013)	0.001		2.663 (0.956,7.419)	0.062		2.584 (1.769,3.262)	0.003		1.391 (0.203,9.546)	0.741
Pyruvate	17.55	0.519 (0.290,0.928)	0.027		0.528 (0.294,0.947)	0.032		0.391 (0.181,0.845)	0.022		0.486 (0.270,0.886))	0.021		0.179 (0.033,0.977)	0.075
Pipecolate	27.65	0.531 (0.301,0.937)	0.029		0.537 (0.297,0.974)	0.043		0.840 (0.380,1.855)	0.671		0.605 (0.358,1.023)	0.063		0.552 (0.099,3.96)	0.522
Indoleacetate	43.73	1.766 (1.054,2.958)	0.031		1.617 (1.000,2.614)	0.052		1.754 (0.936,3.288)	0.083		1.607 (0.658,3.923)	0.062		1.741 (0.904,3.351)	0.157
Glycodeoxycholate	15.70	0.780 (0.620,0.981)	0.034		0.775 (0.610,0.985)	0.056		0.764 (0.563,1.037)	0.081		0.835 (0.664,1.051)	0.124		0.676 (0.230,1.982)	0.512
Adrenate	32.64	1.859 (1.024,3.376)	0.042		1.875 (1.022,3.440)	0.042		1.570 (0.732,3.371)	0.251		1.868 (1.005,3.474)	0.054		1.921 (0.423,8.630)	0.425
Carnitine	65.99	0.479 (0.235,0.975)	0.042		0.480 (0.241,0.956)	0.045		0.515 (0.160,1.662)	0.272		0.522 (0.267,1.021)	0.067		1.026 (0.109,9.657)	0.981
Phenylacetate	24.02	1.624 (1.015,2.600)	0.043		1.804 (1.142,2.849)	0.016		1.570 (0.732,3.371)	0.251		1.868 (1.005,3.474)	0.054		1.921 (0.423,8.630)	0.425

OR, odds ratio; CI, confidence interval; P, P value; MR, mendelian randomization; MR.RAPS, mendelian randomization robust adjusted profile score; GDM, gestational diabetes mellitus.

We performed reverse MR analysis to assess whether GDM causally affect GM-derived metabolites and none of the methods indicated a causal relationship (Supplementary Table 6). The Cochran's Q test revealed heterogeneity of instrumental variables in serine and adrenate. The MR-Egger intercept and the MR-PRESSO global test suggested the presence of directional horizontal pleiotropy in pyruvate (Supplementary Table 5). The leave-one-out analysis confirmed there are no outlier IVs that would have a significant impact on the result (Supplementary Figure 5).

We performed an MVMR analysis to assess the causal effect of GM-derived metabolites on GDM after confounding factors adjusted. For the protect factor carnitine, after adjusting for BMI (OR, 0.733; 95%CI, 0.320-1.678; *P* = 0.462), alcohol drinking (OR, 0.754; 95%CI, 0.405-1.405; *P* = 0.374) smoking (OR, 0.725; 95%CI, 0.393-1.341; *P* = 0.306), and hypertension (OR, 0.626; 95%CI, 0.331-1.187; *P* = 0.152), the causal effect was no longer significant. Detailed MVMR results for other gut metabolites on GDM were shown in Table 4.

**Table 4:** Multivariable MR analyses of gut microbiota-derived metabolites on GDM after adjusting confounding factors.

**Exposure**	**BMI**			**Alcohol drinking**			**Smoking**			**Hypertension**	
	**OR (95%CI)**	***P***		**OR (95%CI)**	***P***		**OR (95%CI)**	***P***		**OR (95%CI)**	***P***
Serine	5.125(2.142,12.261)	<0.001		3.105(1.443,6.680)	0.004		2.324(1.042,5.184)	0.039		3.348(1.581,7.090)	0.002
Pyruvate	0.424(0.214,0.839)	0.014		0.466(0.294,0.740)	0.001		0.678(0.352,1.309)	0.247		0.492(0.292,0.830)	0.008
Pipecolate	0.542(0.327,0.899)	0.018		0.698(0.423,1.151)	0.159		0.669(0.398,1.123)	0.128		0.648(0.376,1.116)	0.118
Indoleacetate	1.961(0.923,4.164)	0.080		1.684(1.047,2.708)	0.032		1.710(1.029,2.842)	0.039		1.813(1.144,2.875)	0.011
Glycodeoxycholate	0.810(0.551,1.192)	0.285		1.058(0.770,1.454)	0.727		0.893(0.721,1.105)	0.297		0.623(0.556,0.699)	<0.001
Adrenate	2,277(1.686,3.076)	<0.001		1.851(1.270,2.699)	0.001		1.878(1.310,2.692)	0.001		1.829(1.212,2.760)	0.004
Carnitine	0.733(0.320,1.678)	0.462		0.754(0.405,1.405)	0.374		0.725(0.393,1.341)	0.306		0.626(0.331,1.187)	0.152
Phenylacetate	1.184(0.637,2.202)	0.594		1.072(0.647,1.773)	0.778		1,791(1.372,2.339)	0.000		1.596(1.154,2.208)	0.005

OR, odds ratio; CI, confidence interval; P, P value; MR, mendelian randomization; GDM, gestational diabetes mellitus; BMI, body mass index.

The metabolic pathway analysis shown that “Phenylalanine metabolism” and “Citrate cycle (TCA cycle)” pathways are associated with the risk of GDM (Supplementary Table 7).

## DISCUSSION

In this bidirectional MR study, we detected causal associations between specific bacterial genera and the risk of GDM. Accumulating evidence has shown significant dysbiosis of the gut microbiota in pregnant women with impaired glucose tolerance, which may contribute to the development of GDM. The gut bacteria associated with increased risk of GDM included *Collinsella* [21], *Olsenella* [22], *Prevotella9* [23], *Lachnoclostridium* [24], and *Ruminococcus2* [22]. While, beneficial butyrate-producing bacteria, such as *Oscillibacter* [22] and *Methanobrevibacter* [25] were found to have a protective effect on GDM. These results from epidemiology were consistent with our study. Specifically, our MR analysis found *Collinsella* is positively correlated with GDM. This association remained statistically significant even after adjusting for multiple comparisons and controlling for covariates such as BMI, alcohol drinking, smoking, and hypertension. Similar with our results, Zhang et al. and Zhong et al. reported an enrichment of *Collinsella* and its species *Collinsella intestinalis* in fecal samples from GDM pregnancies [10, 26]. Interestingly, another case-control study demonstrated the enrichment of *Collinsella* in GDM patients last to postpartum, suggesting its potential contribution to the long-term risk of type 2 diabetes [22]. Meanwhile, population-based studies have consistently reported a higher abundance of the *Collinsella* genus in individuals with type 2 diabetes, atherosclerosis, rheumatoid arthritis, and overweight individuals [26–28] as well as a positive correlation of the *Collinsella* with serum cholesterol was detected by mice model [29]. *In vitro* experiments have shown that *Collinsella* reduces the expression of the ZO-1 tight junction protein, thereby impairing the integrity of the intestinal barrier [28]. Increased gut permeability allows higher levels of lipopolysaccharide, produced by gut microbiota, to enter the bloodstream [30], which can lead to systemic inflammation. This may explain the potential mechanism through which *Collinsella* contributes to the development of these diseases [31]. All the evidence above suggests that the *Collinsella* could potentially serve as a novel target for the prevention and treatment of the aforementioned diseases. However, further functional experiments and RCTs are required to support this finding.

Regarding to gut metabolites, in this study, MR results showed suggestive evidence of genetically increased phenylacetic acid (PA) with a higher risk of GDM as well as the metabolic pathway analysis revealed that “Phenylalanine metabolism” pathway is closely related to GDM. In a nested case-control study involving 105 women in early pregnancy, it was found that GDM patients had significantly higher levels of PA compared to the control group [32]. This finding is consistent with previous research showing elevated levels of PA in patients with impaired fasting glucose, even after accounting for traditional risk factors [33]. PA is an organic compound primarily produced through microbial phenethylamine metabolism by bacteria [34, 35]. Once absorbed into the portal system, PA can be converted by the liver into phenylacetylglutamine (PAGln) [36]. A cohort study involving 1,797 female twins demonstrated an association between PAGln and the expression of the cell death activator CIDE-C, which plays a role in regulating insulin resistance in adipose tissue [37]. The expression of microbial PAGln-synthesis related enzyme genes was positively correlated with the absolute count of neutrophils, indicating systemic inflammation [38], and evidence indicated that inflammation status can trigger the onset of hyperglycemia [39]. Collectively, these findings provided evidence that PA and the "Phenylalanine metabolism" pathway may play a significant role in the pathogenesis of GDM. Additionally, we found a positive association between indole acetate and GDM. Zhu et al. developed a multi-metabolite model that accurately predicted the risk of GDM, including indole acetate [40], which supported our result.

Carnitine, a quaternary ammonium compound abundant in red meat [41] can be converted into trimethylamine-N-oxide (TMAO) through a microbiota-dependent mechanism [42]. Cellular and *in vivo* experiments supported the role of TMAO in inhibiting gluconeogenesis and increasing blood glucose by blocking the hepatic insulin signaling pathway [43, 44]. However, evidence from human studies is not always consistent. Several observational studies have suggested an increased risk of diabetes with elevated TMAO levels [45, 46], whereas cohort study and MR analysis indicated no association [19, 47]. Interestingly, we identified a protective effect of carnitine on GDM, which is supported by a prospective cohort study in China. This study found an inverse relationship between the concentration of L-carnitine and the risk of GDM, with a clear threshold effect [48]. However, in a birth cohort study conducted in Boston, the concentration of carnitine and other precursors of TMAO showed no association with GDM [49]. Additionally, our further multivariable MR analysis found no direct causal effect between carnitine and GDM after confounders adjusted. Considering the unclear mechanism, inconsistent results, and potential confounding factors, the causal relationship between carnitine and the risk of GDM should be interpreted with caution.

Our study has several strengths. Firstly, it is the first MR analysis to explore the possible causal associations among GM, GM-derived metabolites, and GDM. Secondly, the exposure and outcome data are derived from the largest GWAS conducted to date. Furthermore, we employed bidirectional MR, multivariable MR, and several sensitivity analyses, which enhance the robustness of our findings. Thirdly, confounding variables and reverse causation are less likely to have an impact on the causal associations. Therefore, our study may offer potential gut biomarkers that can be further investigated in functional studies related to GDM.

Apparently, there are still some limitations. Firstly, we set the significance threshold of exposure instrumental variables (IVs) at 1e-05 due to the limited number of IVs meeting genome-wide significance criteria. However, we tested the *F*-statistics to avoid the weak instrumental bias. Secondly, the original GWAS population is predominantly of European descent, thus limiting the generalizability of our findings to other ethnicities. Thirdly, due to the limited resolution of 16S rRNA sequencing, our MR analyses were performed at the bacterial genus level rather than at a more specific species level. Finally, although GM and GM-derived metabolites may be influenced by dietary habits, we were unable to account for these confounding factors in the multivariable MR analysis due to the lack of publicly available GWAS on dietary habits.

In conclusion, our study employed bidirectional MR analyses on GWAS summary data to comprehensively investigate the causal effects of gut microbiota and gut microbiota-derived metabolites on GDM. Our findings offer valuable insights into the mechanisms of GDM and may contribute to the development of prevention and treatment strategies targeting gut biomarkers. However, further studies are needed to validate these results.

## MATERIALS AND METHODS

### Data sources

The GM dataset conducted by the Microbiome Genome (MiBioGen) consortium consists of 24 multiple ancestry cohorts including 18,340 subjects [50]. After extracting DNA from fecal samples, data was generated by the Illumina platform. Setting SILVA database as the reference, 16S rRNA gene sequencing pipeline was conducted to profile the microbial composition [51], with the annotation to genus and higher level.

Genetic variants for gut metabolites were collected from a pooled dataset of 7,824 European ancestry participants (TwinsUK and KORA cohorts), which tested 486 metabolite concentrations after sex and age corrected [52]. Then we manually checked HMDB database to obtain a list of 81 GM-derived metabolites (i.e., butyric acid, choline, glutamate, kynurenine, tyrosine) from all the quantified metabolites in the GWAS which includes summary data [53].

GWAS summary statistics for GDM was extracted from the FinnGen consortium included 123,579 female subjects (5,687 cases and 117,892 controls) [54]. These individuals were genotyped using Illumina and Affymetrix chips arrays, and 16,379,784 variants were analyzed in total. Association analysis was conducted with sex, age, genotyping batch, and 10 principal components as covariates. Details of GM, GM-derived metabolites, and GDM GWAS datasets used in this study were listed in Supplementary Table 8.

### Instrumental variables

Five steps were applied to select the optimal IVs: (1) SNPs under a locus-wide significance threshold of *P* < 1e-05 were obtained as potential IVs related to each exposure traits, respectively [18]. (2) Linkage disequilibrium (LD) based clumping was performed to ensure the potential IVs are independent (*r^2^* < 0.001, window size = 10,000 kb) [55]. (3) SNPs with minor allele frequency < 0.01 and palindromic SNPs were excluded. (4) The proxy SNPs (*r^2^* > 0.8) were selected based on European population data in the 1000 Genome project after removing the SNPs closely related to the outcome phenotype (*P* < 5e-08) [56]. (5) SNPs with *F*-statistics < 10 were excluded to avoid the weak instrumental bias [57].

### Statistical analyses

We used the inverse-variance weighted (IVW) method as the primary MR analysis to detect the causal association between exposure (GM, GM-derived metabolites) and outcome (GDM). The IVW method calculates the total causal effect by using the weighted linear regression model combined with the weight coefficient, under the condition that the intercept is zero [58]. IVW results were corrected for multiple comparisons applying the *q*-value procedure (*q* < 0.1), while *P* < 0.05 but *q* > 0.1 was considered to have a suggestive association [59]. After IVW analysis, GM and metabolites that were found to be causally related to GDM would be selected for further analyses.

Several MR methods including maximum Likelihood (MaxLik), weighted median (WM), MR-Egger regression and MR robust adjusted profile score (MR.RAPS) were also conducted to test the robustness of our study. MaxLik estimates the parameter values that have the greatest likelihood of leading to a particular outcome by using the known sample. Its standard error would be lower than IVW when heterogeneity and horizontal pleiotropy do not exist [60]. WM improves the power of causality detection based on the assumption that up to 50% IVs are valid [61]. MR-Egger regression method could identify and correct pleiotropy, but the estimation accuracy will be very low unless using a larger sample size [62]. MR.RAPS applies robust estimates to correct for systematic and idiosyncratic pleiotropy, the results of which are unbiased even though weak IVs exist [63].

Cochran’s IVW Q statistics and leave-one-out analysis were used to identify potential heterogeneous IVs. MR-Egger intercept and MR Pleiotropy RESidual Sum and Outlier (MR-PRESSO) global test were conducted to test whether directional horizontal pleiotropy is driving the results of MR analyses [64, 65].

Reverse MR analysis was used to confirm the direction of causality. The methods were similar to forward MR, except for setting GDM as the exposure and GM or GM-derived metabolites as the outcomes. Finally, we conducted multivariable MR (MVMR) analysis, taking into account potential confounders that might influence the outcome. Specifically, four confounders including BMI, alcohol drinking, smoking, and hypertension were adjusted in MVMR, respectively.

For GM-derived metabolites that achieved the significant threshold of *P* < 0.05 by IVW method, we used MetaboAnlyst software to conduct the metabolic pathway analysis [66].

**Figure 4:**
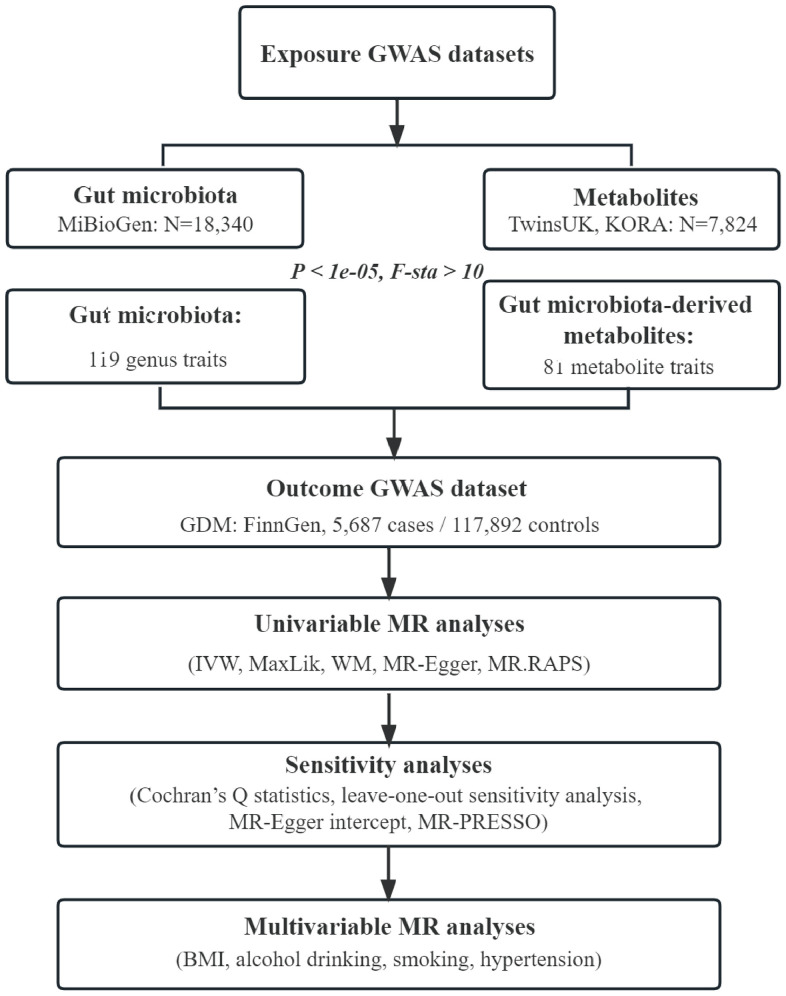
**Flowchart of this study.** GWAS, genome-wide association study; GDM, gestational diabetes mellitus; IVW, inverse-variance weighted; MaxLik, maximum likelihood; WM, weighted median; MR.RAPS, mendelian randomization robust adjusted profile score; MR-PRESSO, mendelian randomization pleiotropy residual sum and outlier; BMI, body mass index.

Flowchart of this study was shown in Figure 4. All MR analyses were performed by the packages “TwoSampleMR”, “MRPRESSO”, and “qvalue” in R software.

### Consent for publication

All the authors endorsed the publication of the manuscript.

## Supplementary Material

Supplementary Figures

Supplementary Table 1

Supplementary Table 2

Supplementary Tables 3-8
