# Supplementary material for: Investigating causal associations among gut microbiota, gut microbiota-derived metabolites, and gestational diabetes mellitus: a bidirectional Mendelian randomization study
Source: Aging (Albany NY). 2023 Aug 23;15(16):8345–66. doi: 10.18632/aging.204973 (PMC10497006; doi:10.18632/aging.204973)
Supplement: Supplementary Figures [file aging-15-204973-s001.pdf]

SUPPLEMENTARY FIGURES

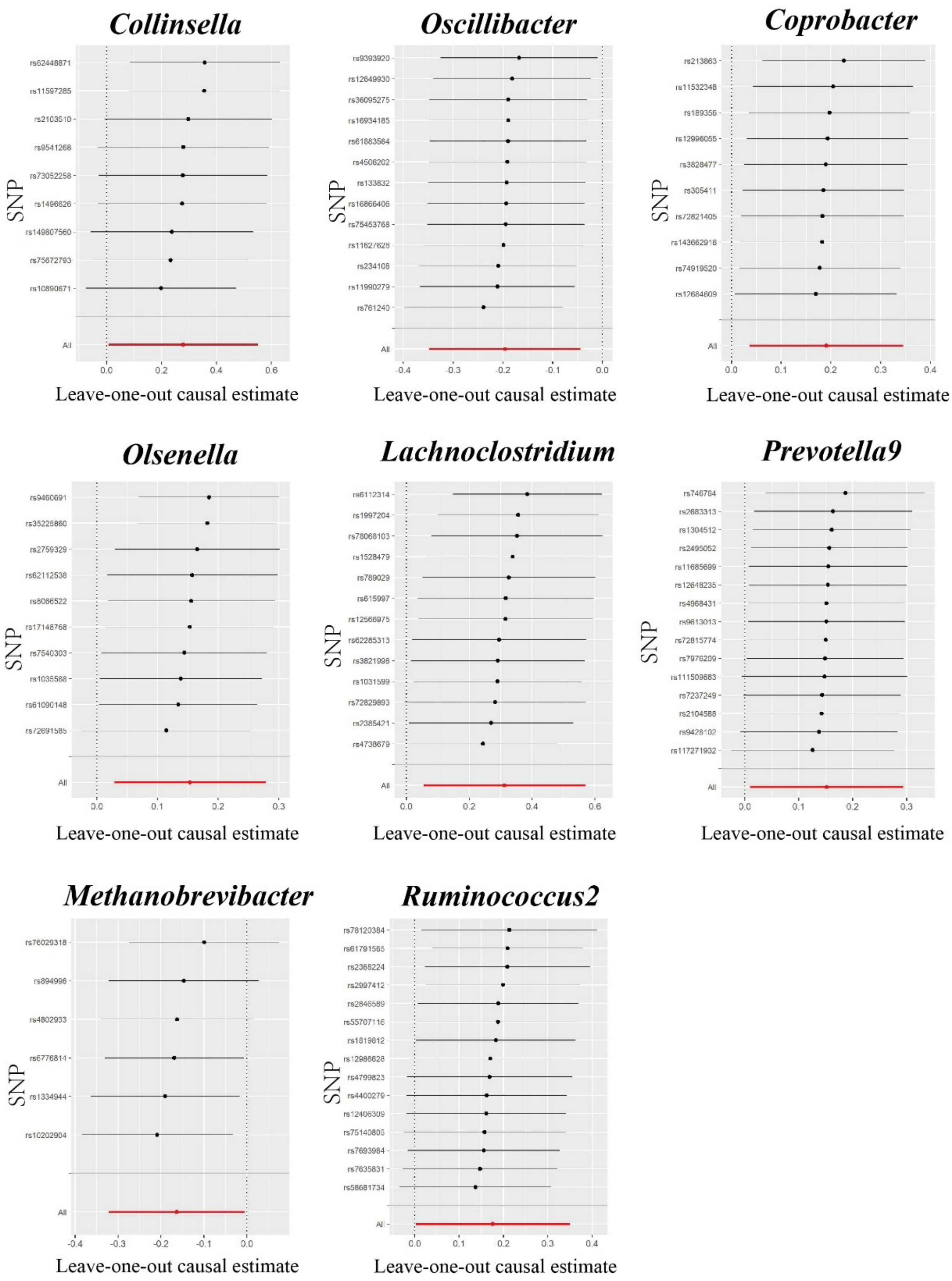

Supplementary Figure 1. Leave-one-out plots for the causal association between gut microbiota and GDM in forward MR analyses.

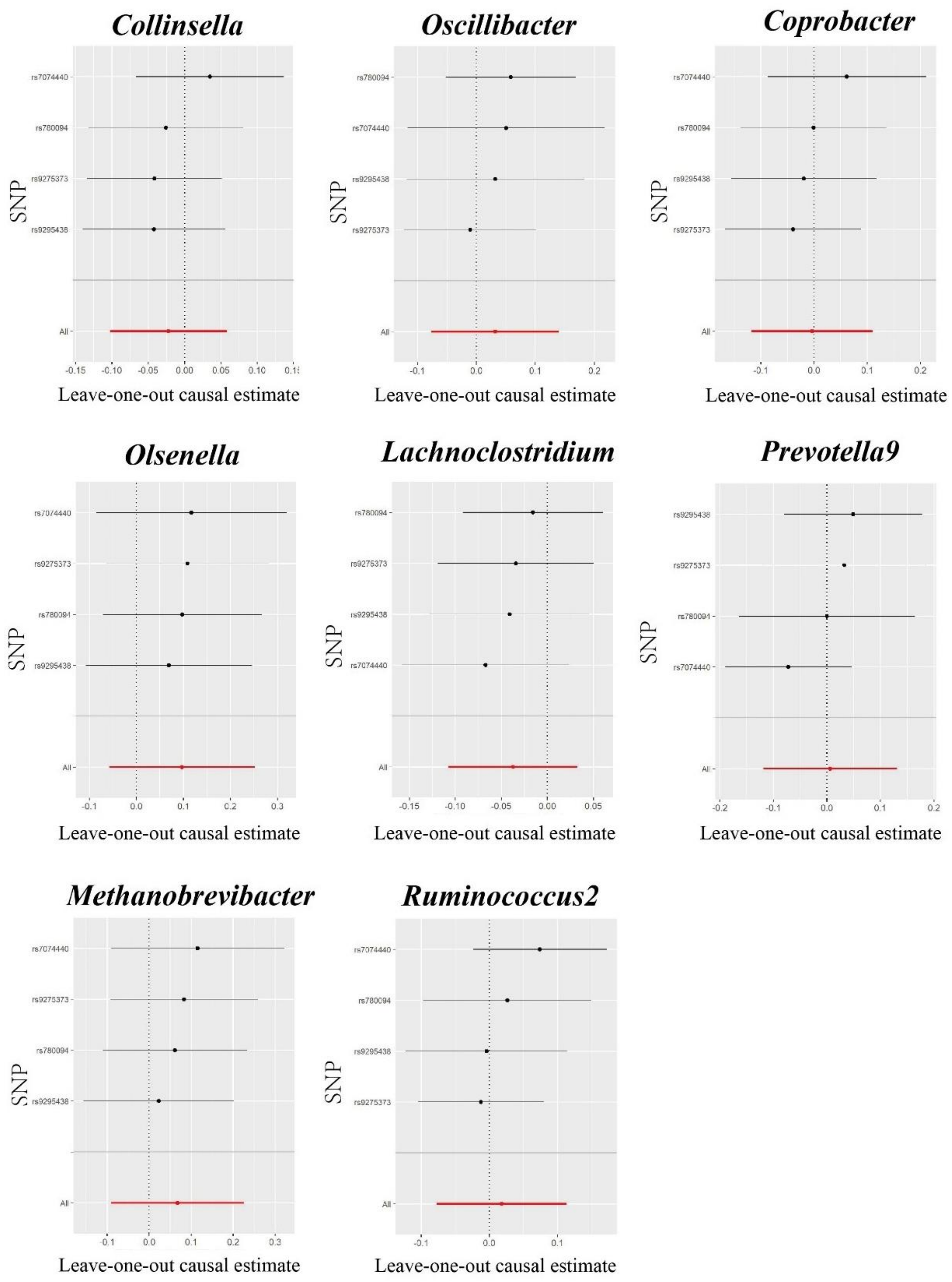

Supplementary Figure 2. Leave-one-out plots for the causal association between GDM and gut microbiota in reverse MR analyses.

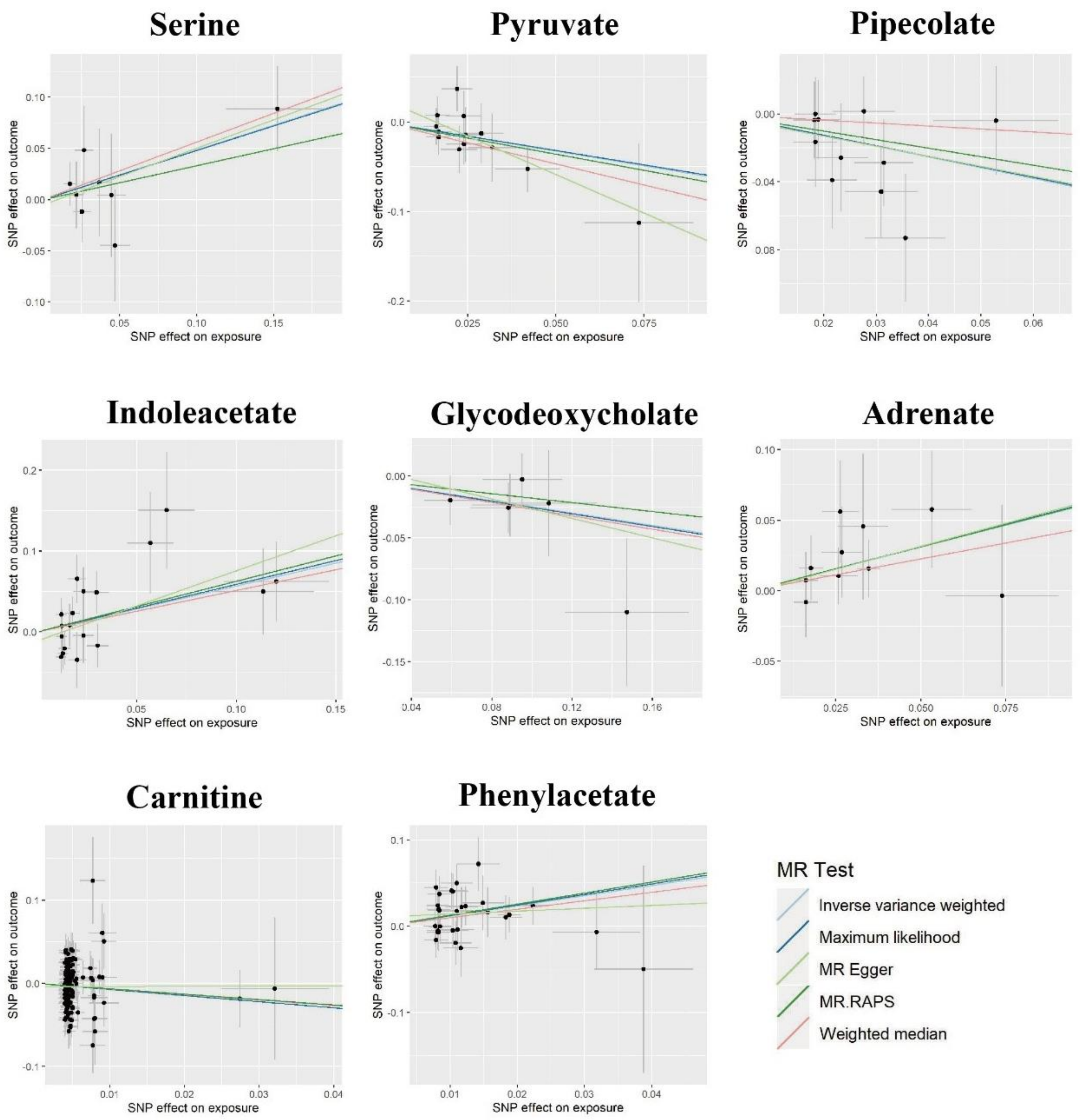

Supplementary Figure 3. Scatter plots for the causal association between gut microbiota-derived metabolites and GDM.

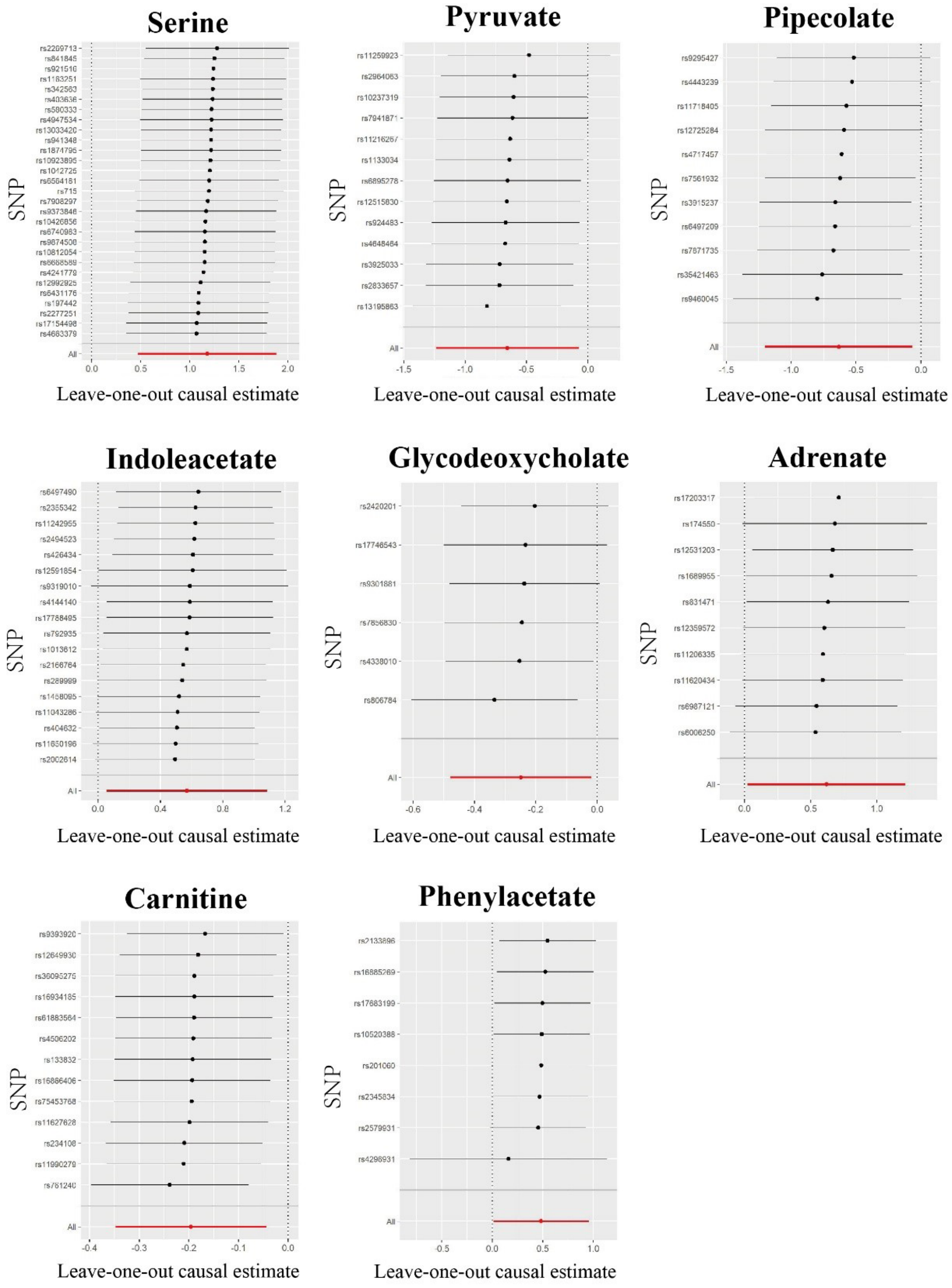

Supplementary Figure 4. Leave-one-out plots for the causal association between gut microbiota-derived metabolites and GDM in forward MR analyses.

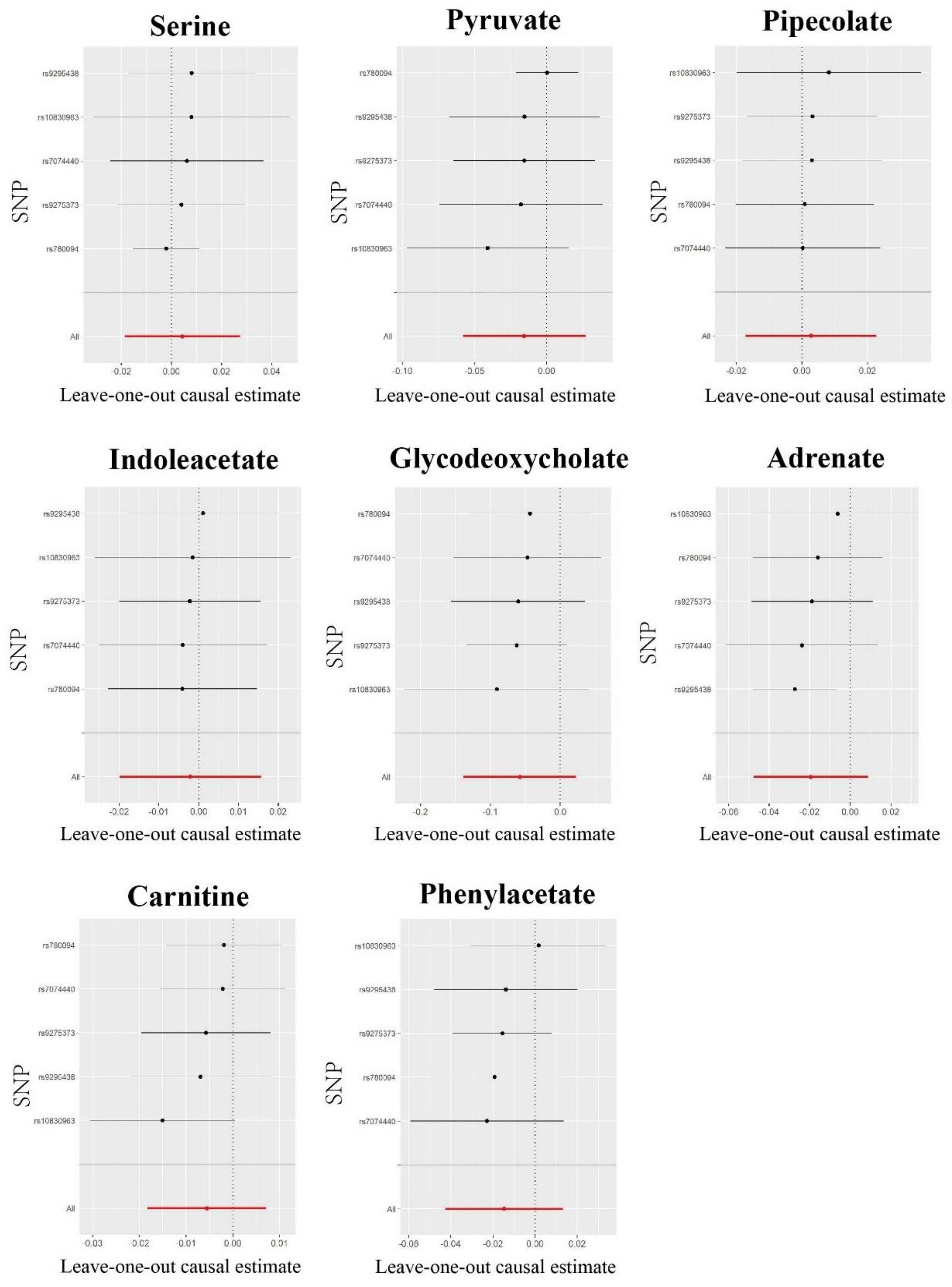

Supplementary Figure 5. Leave-one-out plots for the causal association between gut microbiota-derived metabolites and GDM in reverse MR analyses.
