# Supplementary material for: Investigating causal associations among gut microbiota, gut microbiota-derived metabolites, and gestational diabetes mellitus: a bidirectional Mendelian randomization study
Source: Aging (Albany NY). 2023 Aug 23;15(16):8345–66. doi: 10.18632/aging.204973 (PMC10497006; doi:10.18632/aging.204973)
Supplement: Supplementary Tables 3-8 [file aging-15-204973-s004.pdf]

Supplementary Table 3. Tests for detecting horizontal and directional pleiotropy in forward MR analysis.

Exposure		MR-PRESSO global test		MR-Egger intercept pEgger			Cochran's Q test	
		MR-PRESSO RSSobs	*P* value	Egger-intercept	Standard Error	*P* value	IVW (*P*)	MR-Egger (*P*)
Gut microbiota	*Collinsella*	11.512	0.409	-0.044	0.035	0.252	0.341	0.391
	Oscillibacter	7.355	0.905	-0.025	0.027	0.372	0.903	0.910
	Coprobacter	3.576	0.960	-0.025	0.031	0.445	0.969	0.973
	Olsenella	13.639	0.315	0.003	0.029	0.921	0.261	0.190
	Lachnoclostridium	20.241	0.167	-0.005	0.031	0.855	0.138	0.101
	Prevotella9	6.633	0.977	-0.015	0.021	0.467	0.973	0.972
	Methanobrevibacter	6.006	0.568	0.074	0.043	0.162	0.950	0.901
	Ruminococcus2	17.376	0.363	0.009	0.017	0.597	0.363	0.314
Gut metabolite	Serine	27.181	0.651	0.011	0.012	0.362	0.589	0.583
	Pyruvate	9.313	0.802	0.027	0.021	0.218	0.808	0.874
	Pipecolate	7.775	0.802	-0.001	0.023	0.962	0.807	0.731
	Indoleacetate	25.269	0.175	-0.011	0.011	0.314	0.128	0.137
	Glycodeoxycholate	3.880	0.751	0.013	0.048	0.803	0.758	0.636
	Adrenate	3.852	0.957	-0.001	0.021	0.963	0.954	0.919
	Carnitine	168.085	0.185	-0.004	0.005	0.483	0.174	0.167
	Phenylacetate	5.637	0.803	-0.005	0.017	0.776	0.869	0.798

Supplementary Table 4. Reverse MR analyses of GDM on gut microbiota by different methods.

Outcome	Inverse variance weighted		Maximum likelihood		Weighted median		MR.RAPS		MR Egger	
	β(95%CI)	*P*	*β*(95%CI)	*P*	*β*(95%CI)	*P*	*β*(95%CI)	*P*	*β*(95%CI)	*P*
Collinsella	-0.022 (-0.103,0.058)	0.592	-0.023 (-0.102,0.057)	0.581	0.004 (-0.100,0.107)	0.941	-0.035 (-0.089,0.019)	0.203	-0.38 (-0.431,0.355)	0.872
Oscillibacter	-0.004 (-0.116,0.111)	0.951	-0.04 (-0.118,0.111)	0.950	-0.014 (-0.157,0.128)	0.842	0.049 (-0.029,0.127)	0.221	0.033 (-0.466,0.533)	0.913
Coprobacter	-0.037 (-0,107,0.033)	0.302	-0.038 (-0.109,0.033)	0.290	-0.023 (-0.109,0.063)	0.600	-0.009 (-0.058,0.039)	0.700	0.153 (0.126,0.433)	0.390
Olsenella	0.068 (-0.090,0.225)	0.401	0.068 (-0.09,0.226)	0.400	0.005 (-0.183,0.193)	0.962	0.055 (-0.053,0.162)	0.320	0.048 (-0.579,0.676)	0.890
Lachnoclostridium	0.097 (-0.058,0.252)	0.222	0.096 (-0.058,0.253)	0.221	0.077 (-0.107,0.261)	0.410	0.008 (-0.098,0.114)	0.881	0.126 (-0.490,0.742)	0.731
Prevotella9	0.032 (-0.076,0.139)	0.564	0.032 (-0.069,0.133)	0.532	0.016 (-0.104,0.135)	0.798	0.002 (-0.066,0.070)	0.964	0.260 (-0.150,0.670)	0.340
Methanobrevibacter	0.006 (-0.119,0.131)	0.920	0.007 (-0.087,0.100)	0.893	0.020 (-0.102,0.142)	0.753	0.027 (-0.036,0.090)	0.403	-0.086 (-0.684,0.511)	0.800
Ruminococcus2	0.018 (-0.078,0.113)	0.710	0.018 (-0.058,0.046)	0.642	-0.021 (-0.119,0.077)	0.680	-0.006 (-0.058,0.046)	0.826	0.111 (-0.337,0.558)	0.676

Supplementary Table 5. Tests for detecting horizontal and directional pleiotropy in reverse MR analysis.

Exposure		MR-PRESSO global test		MR-Egger intercept pEgger			Cochran's Q test	
		MR-PRESSO RSSobs	*P* value	Egger-intercept	Standard error	*P* value	IVW (*P*)	MR-Egger (*P*)
Gut microbiota	*Collinsella*	7.280	0.377	0.003	0.037	0.943	0.366	0.206
	Oscillibacter	5.403	0.432	-0.043	0.038	0.377	0.324	0.348
	Coprobacter	5.260	0.512	-0.007	0.047	0.895	0.485	0.298
	Olsenella	0.794	0.935	-0.005	0.058	0.934	0.932	0.807
	Lachnoclostridium	4.161	0.564	-0.036	0.026	0.301	0.503	0.802
	Prevotella9	12.033	0.235	0.017	0.056	0.783	0.136	0.071
	Methanobrevibacter	2.462	0.730	0.003	0.059	0.956	0.736	0.531
	Ruminococcus2	9.898	0.266	-0.017	0.042	0.715	0.189	0.112
Gut metabolite	Serine	306.675	0.049	0.009	0.006	0.217	0.002	0.026
	Pyruvate	-29.025	0.001	-0.022	0.005	0.028	0.001	0.714
	Pipecolate	1.836	0.882	0.004	0.006	0.548	0.873	0.856
	Indoleacetate	1.764	0.887	0.003	0.005	0.635	0.852	0.783
	Glycodeoxycholate	6.721	0.446	-0.024	0.023	0.357	0.279	0.302
	Adrenate	15.901	0.172	-0.001	0.009	0.973	0.034	0.015
	Carnitine	37.744	0.076	-0.005	0.002	0.115	0.061	0.073
	Phenylacetate	11.931	0.257	0.011	0.007	0.208	0.137	0.287

Supplementary Table 6. Reverse MR analyses of GDM on gut metabolites by different methods.

Outcome	Inverse variance weighted		Maximum likelihood		Weighted median		MR.RAPS		MR Egger	
	β(95%CI)	*P*	*β*(95%CI)	*P*	*β*(95%CI)	*P*	*β*(95%CI)	*P*	*β*(95%CI)	*P*
Serine	0.004 (-0.019,0.027)	0.711	0.005 (-0.007,0.016)	0.438	0.001 (-0.012.0.013)	0.934	0.005 (-0.007,0.016)	0.430	-0.034 (-0.085,0.018)	0.290
Pyruvate	-0.016 (-0.058,0.027)	0.470	-0.017 (-0.037,0.004)	0.120	-0.002 (-0.026,0.022)	0.880	-0.017 (-0.036,0.003)	0.107	0.080 (0.029,0.132)	0.054
Pipecolate	0.003 (-0.017,0.023)	0.790	0.003 (-0.017,0.023)	0.790	-0.001 (-0.023,0.022)	0.960	0.003 (-0.017,0.023)	0.790	-0.014 (-0.066,0.038)	0.640
Indoleacetate	-0.002 (-0.020,0.016)	0.810	-0.002 (-0.020,0.016)	0.810	-0.001 (-0.021,0.019)	0.930	-0.002 (-0.020,0.016)	0.820	-0.014 (-0.061,0.033)	0.610
Glycodeoxycholate	-0.058 (-0.138,0.023)	0.160	-0.058 (-0.130,0.014)	0.113	-0.052 (-0.132,0.027)	0.200	-0.058 (-0.131,0.015)	0.117	0.042 (-0.155,0.240)	0.703
Adrenate	-0.019 (-0.048,0.009)	0.177	-0.020 (-0.038,-0.002)	0.029	-0.023 (-0.044,-0.003)	0.026	-0.020 (-0.042,0.001)	0.057	-0.018 (-0.104,0.068)	0.708
Carnitine	-0.006 (-0.018,0.007)	0.390	-0.006 (-0.012,0.000)	0.059	-0.003 (-0.011,0.005)	0.495	-0.006 (-60.766,90.754)	0.053	0.016 (-0.005,0.037)	0.236
Phenylacetate	-0.015 (-0.043,0.013)	0.300	-0.015 (-0.036,0.006)	0.170	-0.020 (-0.048,0.007)	0.150	-0.015 (-0.037,0.007)	0.170	-0.063 (-0.126,0.001)	0.150

Supplementary Table 7. Metabolic pathway associated with GDM.

Metabolic pathway	Involved metabolites	*P* value	Database
Phenylalanine metabolism	Phenylacetic acid	0.026	KEGG SMP
Citrate cycle (TCA cycle)	Pyruvate	0.041	KEGG SMP
Pyruvate metabolism	Pyruvate	0.056	KEGG SMP
Lysine degradation	L-Pipecolate	0.063	KEGG SMP
Glycolysis / Gluconeogenesis	Pyruvate	0.065	KEGG SMP SMP
Alanine, aspartate and glutamate metabolism	Pyruvate	0.070	KEGG SMP SMP SMP
Glyoxylate and dicarboxylate metabolism	Pyruvate	0.080	KEGG
Glycine, serine and threonine metabolism	Pyruvate	0.083	KEGG SMP
Cysteine and methionine metabolism	Pyruvate	0.083	KEGG SMP SMP
Arginine and proline metabolism	Pyruvate	0.095	KEGG SMP
Tryptophan metabolism	Indole-3-acetate	0.102	KEGG SMP
Tyrosine metabolism	Pyruvate	0.104	KEGG SMP SMP

Supplementary Table 8. Detailed information for genome-wide association studies involved in the present Mendelian randomization study.

Variable	Consortium or study	Sample size	Journal	Year	Cohort	Nation of cohort	Number of samples
**Gut microbiota**	MiBioGen	18,340	Nat Genet.	2021	BSPSPC	Germany	721
					CARDIAw	USA	257
					COPSAC	Denmark	380
					DanFunD16	Denmark	2,396
					FGFP	Belgian	2,259
					FOCUS	Germany	960
					GEM_HCE_v12	Canada	378
					GEM_HCE_v24	Canada	203
					GEM_ICHIP_HCE	Canada	662
					GenR	The Netherlands	1,328
					HCHS/SOL	USA	1,097
					KSCS	South Korea	811
					LLD	The Netherlands	875
					METSIM	Finland	522
					MIBS	The Netherlands	80
					NGRC	USA	77
					NTR	The Netherlands	279
					PNP	Israel	481
					POPCOL	Sweden	134
					RS3	The Netherlands	1,220
					SHIP	Germany	996
					SHIP-TREND	Germany	905
					TwinsUK	UK	1,205
**Gut metabolites**	TwinsUK, KORA F4	7,824	Nat Genet.	2017	KORA F4	Germany	1,768
					TwinsUK	UK	6,056
**BMI**	UK Biobank	461,460		2018	UK Biobank	European	461,460
**alchol drinking**	UK Biobank	462,346		2018	UK Biobank	European	462,346
**smoking**	GSCAN	607,291		2019	GSCAN	European	311,629 cases/321,173 controls
**hypertension**	UK Biobank	462,346		2018	UK Biobank	European	462,346
**GDM**	FinnGen	123,579		2021	FinnGen	European	5,687 cases/117,892 controls
